# Herpes Simplex Virus-2 Meningoencephalitis With Abducens Nerve Palsy With Literature Review

**DOI:** 10.7759/cureus.15523

**Published:** 2021-06-08

**Authors:** Sachin Patil, Phillip Beck, Taylor B Nelson, Andres Bran, William Roland

**Affiliations:** 1 Infectious Disease, University of Missouri, Columbia, USA

**Keywords:** herpes simplex virus, meningitis, encephalitis, abducens nerve palsy, cranial nerve palsy

## Abstract

Herpes simplex virus (HSV), a human alpha herpes virus, is responsible for most infections caused by herpes viruses worldwide. Among the herpes simplex viruses, both HSV 1 and 2 cause significant morbidity. HSV-2 accounts for most genital infections with extragenital complications involving the groin, thigh, or other pelvic areas. HSV-2 is the leading viral cause of sexually transmitted diseases. Viral dissemination via the blood or the cutaneous route during primary infection can affect joints, liver, lungs, spinal cord, and brain. HSV-2, by nature of its higher reactivation frequency, leads to clinical reactivation or subclinical shedding, resulting in increased transmission risk during unprotected sexual encounters. HSV-2 reactivation can result in lesions involving the fingers, skin, eyes, brain, and visceral organs such as the esophagus, lung, and liver. Ocular involvement results in keratitis, blepharitis, conjunctivitis, and rarely necrotizing retinitis. Oculomotor cranial nerve involvement by HSV is a rare entity even in patients with human immunodeficiency virus infection. Clinical features associated with reactivation are seen in primary infections, especially in children and adolescents. A medical literature search resulted in a few cases caused by a varicella-zoster virus but none by HSV. Here we describe a young female with a newly diagnosed meningoencephalitis and abducens nerve palsy (first case) due to a primary HSV infection. She came to the emergency department with headache, confusion, abnormal behavior and later developed diplopia as an inpatient. She was treated successfully with two weeks of acyclovir.

## Introduction

Herpes simplex viruses (HSV 1 & 2) are deoxyribonucleic acid viruses belonging to the human herpesvirus family's alpha herpesvirus group. Humans are the only known natural reservoir with no known animal vectors. HSV-1 infection acquisition is earlier and frequent than HSV-2 based on epidemiological data [1]. HSV-2 infection is acquired frequently during puberty due to sexual activity initiation, whereas HSV-1 infection occurs commonly during the first decade of life. Risk factors for HSV-2 infection include human immunodeficiency virus (HIV)-infected patients, female sex workers, and men having sex with men [1]. HSV transmission results from close mucocutaneous contact with a patient who has recurrent exuviation with or without symptoms. The virus is not viable at room temperature making transmission via fomites and aerosols rare [1]. Females compared to males have an increased likelihood of acquiring HSV-2 infection and manifesting symptoms. Prior HSV-1 infection does not decrease HSV-2 infection risk but does increase asymptomatic seroconversion [2]. Viral entry via the skin and mucous membranes is followed by neuronal infection and latent infection of the neuronal ganglia. HSV-2 targets the sacral nerve root ganglia, whereas HSV-1 infects the trigeminal ganglia. Both viruses cause a primary infection followed by latency and then clinical recurrences or subclinical shedding depending on the host's immune competence. Reactivation results in centrifugal migration of infectious virions to the peripheral sensory nerves causing vesicular crops in a dermatomal distribution [3]. Clinical manifestations of HSV-2 reactivation (Mollaret meningitis) are different in comparison to the primary infection (acute viral encephalitis). Here we report the first case of a young immunocompetent female presentation with acute left abducens nerve palsy and meningoencephalitis due to a primary HSV-2 infection.

## Case presentation

A 19-year-old white female came to the emergency department (ED) with altered mentation and abnormal behavior. Three days earlier, she developed headache, malaise, nausea, confusion with fever, behavioral changes, and agitation, which prompted her parents to bring her to the ED for evaluation. Her medical history was relevant for anxiety, depression, and obesity. She endorsed social alcohol consumption and routine recreational marijuana use. She denied any intravenous drug abuse and reported being sexually active. At the ED, she had stable vital signs. Clinical exam revealed a young obese female with somnolence, intermittent agitation, a Glasgow coma score of seven, spontaneous limb movements with no nuchal rigidity. Ocular examination revealed a left-sided esotropia. The genital examination was negative for lesions indicative of sexually transmitted disease.

**Figure 1:**
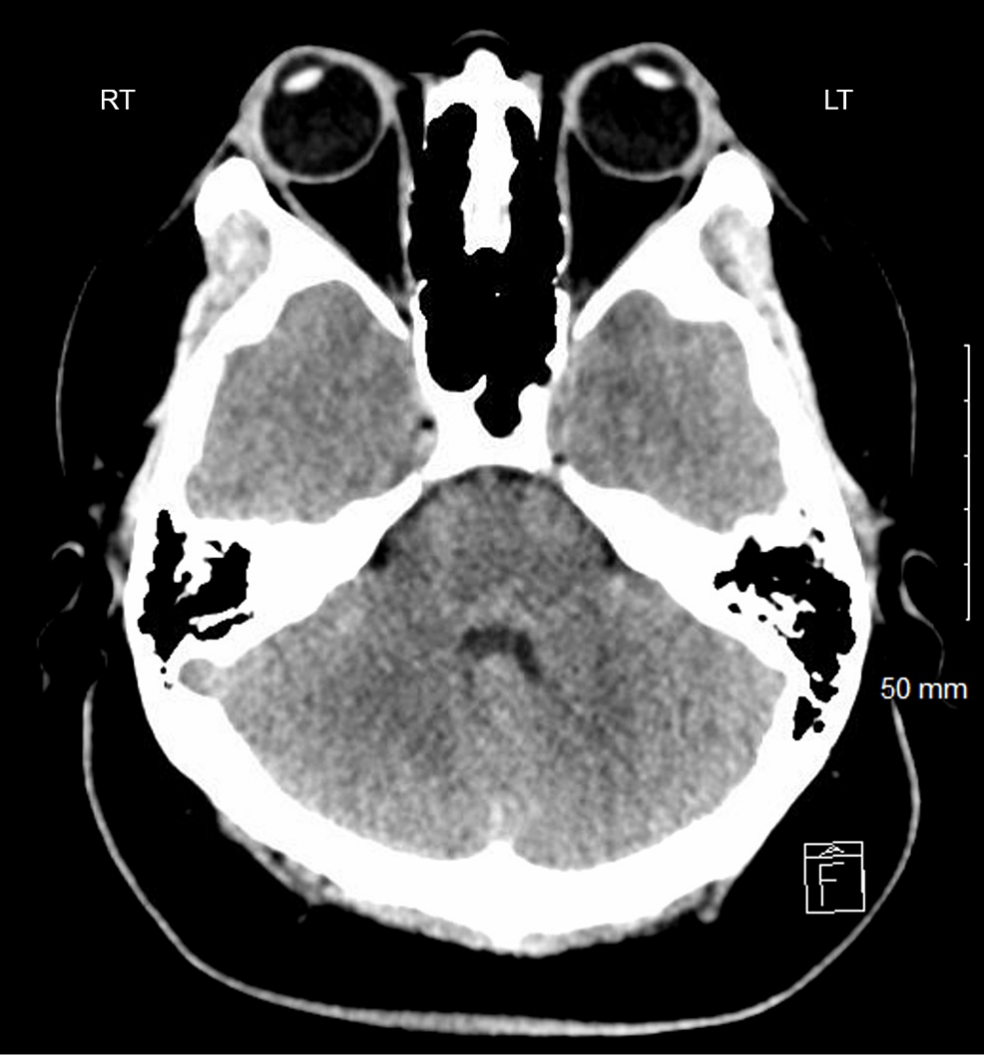
Normal axial CT scan at the level of the fourth ventricle

Comprehensive laboratory workup revealed a normal metabolic panel and blood count revealing a normal white cell count with neutrophilia (absolute granulocyte count of 9.02 x 10^9^/L), lymphopenia (absolute lymph count of 0.59 x 10^9^/L). Lactic acid was increased with a normal creatinine kinase, thyroid-stimulating hormone, and troponin. Urinalysis was not indicative of urinary infection. The testing for rapid influenza A/B and SARS-CoV-2 nasopharyngeal polymerase chain reaction (PCR) was negative. The urine drug screen revealed cannabinoids (Table 1). Chest X-ray revealed no acute cardiopulmonary abnormality (Table 1). Sedatives and antipsychotics were required to control her agitation in the ED. Computed tomography of the head was done, which was normal (Figure 1). Due to her restlessness, fundoscopy could not be performed. Her clinical course was concerning for meningitis/encephalitis, so she underwent a lumbar puncture for cerebrospinal fluid (CSF) and two blood culture sets. The CSF opening pressure was 30 centimeters (cm) of water. Immediately after obtaining the CSF, empiric antibiotics (vancomycin, ceftriaxone), acyclovir, and steroids were initiated. CSF analysis was suggestive of a viral infection with a negative CSF Bio fire PCR and cryptococcal antigen test (Table 1). The empirical regimen was continued, and she was admitted to internal medicine service.

**Table 1:** A summary of laboratory and imaging findings COVID-19 = Coronavirus disease 2019, CSF = Cerebrospinal Fluid, VDRL = Venereal Disease Research Laboratory Test, PCR = Polymerase chain reaction.

Laboratory test/Others	Result	Laboratory test/Imaging	Result
Temperature max	39.5°C	CSF Color	Clear
Complete Blood count	Hemoglobin 11.1 g/dL, Platelet count 263,000/mL	CSF white cell count	808
Metabolic Panel	normal	CSF Neutrophil %	12%
Hemoglobin A1c	5.5% (4 – 6)	CSF Monocytes %	36%
Lactic acid	3.3 mmol/L (0.5 – 2.2)	CSF Lymphocytes %	52%
Creatinine Kinase	50 units/L (20 – 180)	CSF Red Blood Cell count	182
Lipid Panel	Within Normal Limits	CSF VDRL	Negative
Troponin T	˂ 0.01 ng/mL	CSF Cryptococcal Antigen test	Negative
Thyroid-stimulating hormone level	3.290 mIU/L (0.27 – 4.2)	CSF Culture	Negative
Urine Analysis	Negative for UTI	CSF Bio Fire PCR Panel	Negative
Urine Drug Screen	Positive for cannabinoids	CSF HSV 1 & 2 DNA PCR	Positive for HSV-2 DNA
Nasopharyngeal COVID-19 PCR	Negative	Blood Culture	Negative
Nasopharyngeal Influenza A & B Antigen Testing	Negative	Serum Pregnancy test	Negative
HIV 1 & 2 Antigen Antibody testing	Negative	Serum Ferritin	65.2 ng/mL (13 – 150)
Syphilis IgG and IgM testing	Negative	Serum Iron	14 mcg/dL (37 – 145)
TB Gold QuantiFERON	Negative	Chest X-ray	No acute abnormality
Ehrlichia chaffeensis / canis / ewingii CSF and Blood PCR	Negative	Computed Tomography of Head	No acute intracranial abnormality
Anaplasma Serology	Negative	Magnetic Resonance Imaging of Brain	No acute intracranial abnormality

**Figure 2:**
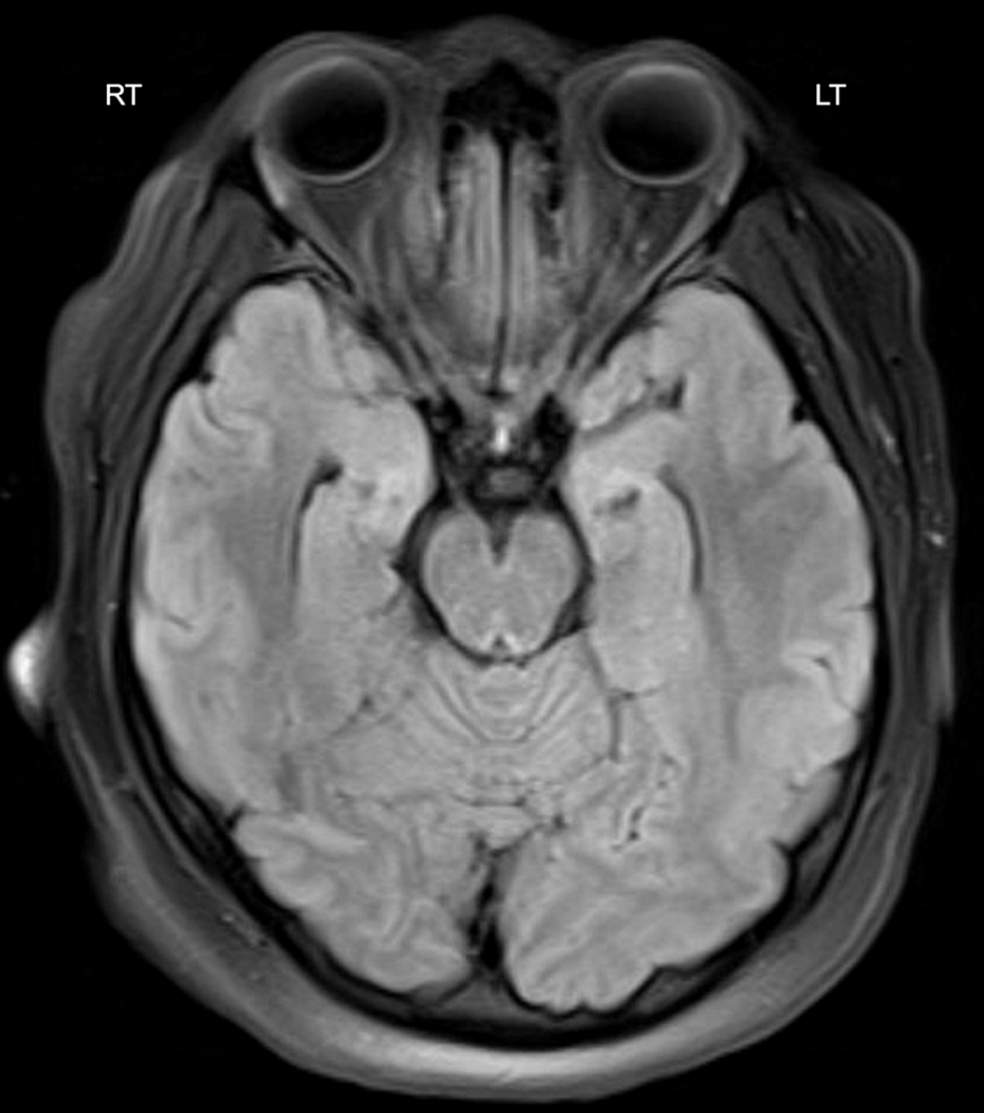
Normal axial flair T2-weighted MRI at the level of inferior colliculus

On day 2 of her hospitalization, she was febrile and encephalopathic with a leukocytosis of 17,550/mL. The infectious disease (ID) team recommended CSF PCR for HSV, VDRL (Venereal Disease Research Laboratory Test), CSF, and blood serology for Ehrlichia, Anaplasmosis. Doxycycline was added to the empirical regimen. Tuberculosis gold QuantiFERON, serology for HIV, and syphilis were negative. Magnetic resonance imaging of the brain revealed no abnormalities (Figure 2). On day 3, her mentation and leukocytosis improved with new onset of diplopia. She informed the medical team of her unprotected sexual encounter with a new partner three weeks before. On day 4, CSF and blood cultures returned negative. CSF and blood PCR for Ehrlichia, Anaplasmosis were negative. Both vancomycin and doxycycline were stopped. A neurology evaluation confirmed left abducens nerve palsy. By day 6, CSF HSV PCR returned positive for HSV-2 DNA, confirming meningoencephalitis (ME) diagnosis, whereas the CSF VDRL was negative. Ceftriaxone was discontinued, and she was discharged on IV acyclovir to complete two weeks of antiviral therapy. An eye patch over the left eye was recommended to prevent diplopia. At the time of her discharge (day 5), her encephalopathy had resolved.

At two weeks follow-up with primary care, ophthalmology, and occupational therapy, she continued to improve with residual diplopia. She was continued on conservative management with an eye patch and physical therapy. Per ophthalmology, it may take six months for her diplopia to resolve completely. At two months follow-up, her diplopia had improved, and she was cleared for driving with a left eye patch.

## Discussion

HSV-2 infection in adults leads to primary genital herpes followed by dormant infection of the sacral nerve root ganglia [1]. HSV-2 can also infect the orofacial area, especially the tonsils or the pharynx. Infrequently during the primary infection, the extragenital spread via blood or cutaneous route can affect local adjoining areas (uterus, prostate, urethra, thigh) in addition to the brain, spinal cord, lungs, liver, or joints. Neurological manifestations include aseptic meningitis, sacral neuralgia, transverse myelitis. Reactivation after the primary infection (HSV 1 & 2) presents as recurrent lesions involving the skin, finger, brain, eyes, and rarely visceral organs (lung, liver, esophagus). Eye lesions include keratitis, blepharitis, conjunctivitis, chorioretinitis, or necrotizing retinitis. HSV encephalitis causes 10% to 20% of all viral encephalitis cases in the United States of America, but it is still the most common recurrent cause [4]. HSV encephalitis incidence is 2.3 cases per one million persons per year. The age distribution is biphasic, with an increased incidence before 30 years and after age 50 [5]. HSV-1 accounts for most cases (>95%) [6,7]. The pathophysiology of encephalitis is different in young adults in comparison to older adults. In adolescence, primary HSV infection can cause encephalitis via the olfactory neurotropic spread, which manifests as a reactivation in elderly patients. Based on our PubMed literature review, neither HSV primary infection nor reactivation has been reported to involve the ocular cranial nerves three, four, or six.

Abducens nerve palsy (ANP) is the most common oculomotor cranial nerve palsy due to multiple underlying disease processes. The incidence of ANP is 11.3/100,000 persons per year, and the proportion of cases attributed to infection is minimal [8]. To date, no study has determined the exact percentage of an infectious cause of ANP. It can be bilateral or unilateral. The abducens nerve (AN) has the longest anatomic course of all oculomotor cranial nerves, arising from the contralateral abducens nucleus in the dorsocaudal pons. It innervates the ipsilateral lateral rectus muscle and is responsible for eye abduction resulting in an outward gaze. Isolated abducens paralysis is one of the classical false localizing signs due to raised intracranial pressure. Local processes such as trauma, infection, inflammation, and neoplasm-induced raised intracranial pressure can affect AN in its course. AN injury results in ipsilateral abduction impairment, diplopia, and eso-deviation. Infections may be responsible for ANP by one or more of the following pathophysiologic mechanisms: ischemic vascular neuritis, primary cranial neuritis, perineuritis due to adjacent meningeal inflammation, and compressive effect [9]. Apart from the herpes viruses, oculomotor cranial nerve involvement is infrequent among other viral encephalitides [9-13]. Other viral causes of ANP in adults are the West Nile virus, hantavirus, enterovirus, and dengue virus [14-17]. Among the herpes viruses, it is more common with varicella-zoster infections of the brain than with Epstein-Barr virus and cytomegalovirus. Even in patients with varicella-zoster infections, ANP occurring along with meningitis or encephalitis is a rare entity [10].

Our patient was a young immunocompetent adult female not on any immunosuppressive medications. Her presentation was approximately three weeks after unprotected sexual intercourse with no genital complaints or brainstem localizing signs or symptoms. Her ME was likely due to a primary infection than a reactivation. HSV-2 accounts for about 5% of these cases caused by HSV [6]. The CSF opening pressure was 30 cm of H2O, which was high normal (<25 cm of H2O), and this is a normal variation due to her increased BMI of 43 kg/m^2^ [18]. CSF differential count was suggestive of a viral cause, and PCR testing was positive for HSV-2. In cases with higher clinical suspicion, we recommend continuing acyclovir until a reliable HSV PCR is negative. The occurrence of ocular cranial nerve palsy along with ME is rare. An outpatient eye evaluation confirmed left lateral rectus palsy with no papilledema. No HSV ME cases with ANP were revealed on a PubMed literature review, and our case is the first one documented to our knowledge. With unilateral ANP and a normal MRI brain with no significantly raised intracranial pressure, the possible mechanism could be primary cranial neuritis or perineuritis from ME. The presence of residual ANP at two months post-discharge supports this explanation. Had this been due primarily to raised intracranial pressure, ANP should have resolved once the pressure declined. At one- and two-month follow-up she still had residual diplopia and esotropia. The recovery rates for infectious causes are unknown in comparison to non-infectious causes. Information from anecdotal case reports reveal recovery in most cases by six months [9,13-17].

## Conclusions

In younger patients with meningoencephalitis and oculomotor cranial nerve palsy, both HSV and syphilis should be considered in the differential diagnosis. HSV-2 should be suspected even in the absence of genital complaints. CSF PCR assay availability has improved the ability to make rapid diagnoses with no delays in treatment. We suggest continuing acyclovir until a dedicated HSV PCR is negative, as in our case. We agree that MRI is the best imaging modality in younger patients with ME and ANP to rule out other causes such as trauma and tumor. Detailed studies on isolated ANP infectious causes, effective antimicrobial therapy duration, immunomodulatory therapy effect, and recovery rates are recommended to improve future treatment and inform these conditions' prognosis.
